# Impact of organizational and individual factors on patient-provider relationships: A national survey of doctors, nurses and patients in China

**DOI:** 10.1371/journal.pone.0181396

**Published:** 2017-07-28

**Authors:** Ping Zhang, Fang Wang, Yao Cheng, Liu yi Zhang, Bei zhu Ye, Hong wei Jiang, Yi Sun, Xi Zhu, Yuan Liang

**Affiliations:** 1 Department of Social Medicine and Health Management, School of Public Health, Tongji Medical College, Huazhong University of Science and Technology, Wuhan, China; 2 Department of Health Management and Policy, College of public health, the University of Iowa, Iowa City, Iowa, United States of America; University of West London, UNITED KINGDOM

## Abstract

**Objectives:**

To provide an empirical examination of patient–provider relationships (PPR) and its association with organizational and individual factors.

**Methods:**

A national cross-sectional survey was conducted by stratified cluster sampling in 77 hospitals across seven provinces in China between July 2014 and April 2015, involving 3621 doctors, 5561 nurses, and 8022 patients with response rates of 62.93%, 61.16%, and 33.08%, respectively. Self-perceived PPR was the outcome variable. Organizational factors included hospital type (western medicine [WM] and traditional Chinese medicine [TCM] hospital); hospital level (tertiary and secondary hospital); area of specialization (internal medicine and surgery); ratio of doctors (nurses) to ward beds; doctors/nurses’ concerns about performance assessment; and patients’ perceptions of healthcare cost. Individual factors included consultation, listening to patients and socio-demographic factors.

**Results:**

54.6% of doctors, 36.6% of nurses, and 10.2% of patients perceived PPR as poor. Organizational factors independently associated with providers’ perception of poor PPR included hospital type (WM vs TCM: OR = 1.25 [95% CI: 1.06–1.47]) and concerns about performance assessment (high vs low levels: OR = 1.40 [95% CI: 1.14–1.72]) for doctors, and concerns about performance assessment (average vs low levels: OR = 0.79 [95% CI: 0.67–0.93]) for nurses. Those associated with patients’ perception of poor PPR included hospital type (WM vs TCM: OR = 0.63 [95% CI: 0.53–0.74]) and hospital level (tertiary vs secondary: OR = 0.65 [95% CI: 0.51–0.82]). Doctors and nurses reporting listening to patients “frequently” had better perceptions of PPR (OR = 0.46 [95%CI: 0.38–0.56] and 0.49 [95% CI: 0.41–0.59] for doctors and nurses, respectively), as did their patients (OR = 0.24 [95% CI: 0.18–0.31] and 0.54 [95% CI: 0.35–0.84] for doctors and nurses, respectively).

**Conclusions:**

Although our findings require validation in different organizational settings given the likely variability of these associations across systems, our results suggest that implementing moderate levels promoting the level of medical treatment, and broadening doctors/nurses training regarding listening to patients, may benefit to enhance PPR.

## Introduction

Over the past few decades, progress in medical science and technology has made treatments more effective. Despite this, patient–provider relationships (PPR) have increasingly deteriorated around the world [1–5]. In China, the deteriorated PPR has caused a large number of medical disputes between patients and healthcare providers, primarily doctors and nurses, with some extreme cases involving violence towards providers [6]. According to a recent survey of the Chinese Hospital Association, the average number of incidents of violence against providers in public hospitals increased from 20.6 cases/hospital in 2008 to 27.3 cases/hospitals in 2012 [7]. According to China’s National Health and Family Planning Commission, there were about 70,000 medical disputes in 2013, and about 80% of violence against providers took place in tertiary general (public) hospitals [8]. The deteriorated PPR not only negatively affects patient care but also threatens the safety of healthcare staff.

Existing research suggests the decline of PPR in China may have three possible causes. The first is the emergence of the Internet with its myriad health-related websites, giving patients wide-ranging access to sources of medical information, and changing their attitudes towards health, healthcare and healthcare providers [9]. In China, another social factor is the market-oriented reform of medical services [10]. Healthcare staff are now viewed as the supplier of services, with patients as their customers. Relationships are therefore between customer and supplier, or buyer and seller of services, which puts healthcare staff and patients in opposing positions. The second is organizational factors [11], especially profit-driven organizational management and performance assessment linked to the income of health staff [12]. With the market-oriented reform of healthcare services [11, 13], providers are now viewed unfavorably as profit-driven rather than service-driven agent. The third is individual factors, including patient–provider communication, the continuity of care and consultation experience [14, 15], which is most reported among the three aspects. Specifically, length of patient-provider interaction and the manner of providers during the interaction were considered to influence patients’ perception of PPR [16–23] and doctors’ perception of PPR [16, 20–23]. Notably, the vast majority of previous studies have used qualitative methods [16,24,25] and PPR improvement recommendation lack evidentiary support, so empirical analysis of PPR and its association with the above factors is needed, especially involving patients, doctors and nurses simultaneously. Additionally, prior studies have also suffered from examining only a small number of influencing factors (almost no organizational factors involved) and from small sample sizes.

To more fully understand PPR perceived by doctors, nurses, and patients in China as well as its association with organizational and individual factors, we conducted a national survey in 77 general hospitals. The aim of the study is to provide evidence and recommendations to improve PPR combined the organizational and individual perspectives. Additionally, in this period of social transition from planned to market economy, healthcare in China, including the deterioration of PPR, is both complex and important. China’s experience may provide an important reference for both developing and developed countries.

## Methods

### Study design and participants

The study was a stratified cluster sampling survey across the whole of mainland China. Provinces were selected from each geographical area, with two from each of East and West China (Shandong and Jiangsu, and Gansu and Yunnan), and one each from South, Central and North China (Guangdong, Hubei and Beijing metropolis), which have a population of 427.15 million, accounted for 31.88% of the total population of China [26]. With reference to the 2013 and 2014 provincial GDP ranking [27], Guangdong and Beijing metropolis are representative of upper income areas; Gansu and Yunnan are representative of low income areas and the other three (Shangdong, Jiangsu and Hubei) are representative of middle income areas in China.

**Fig 1:**
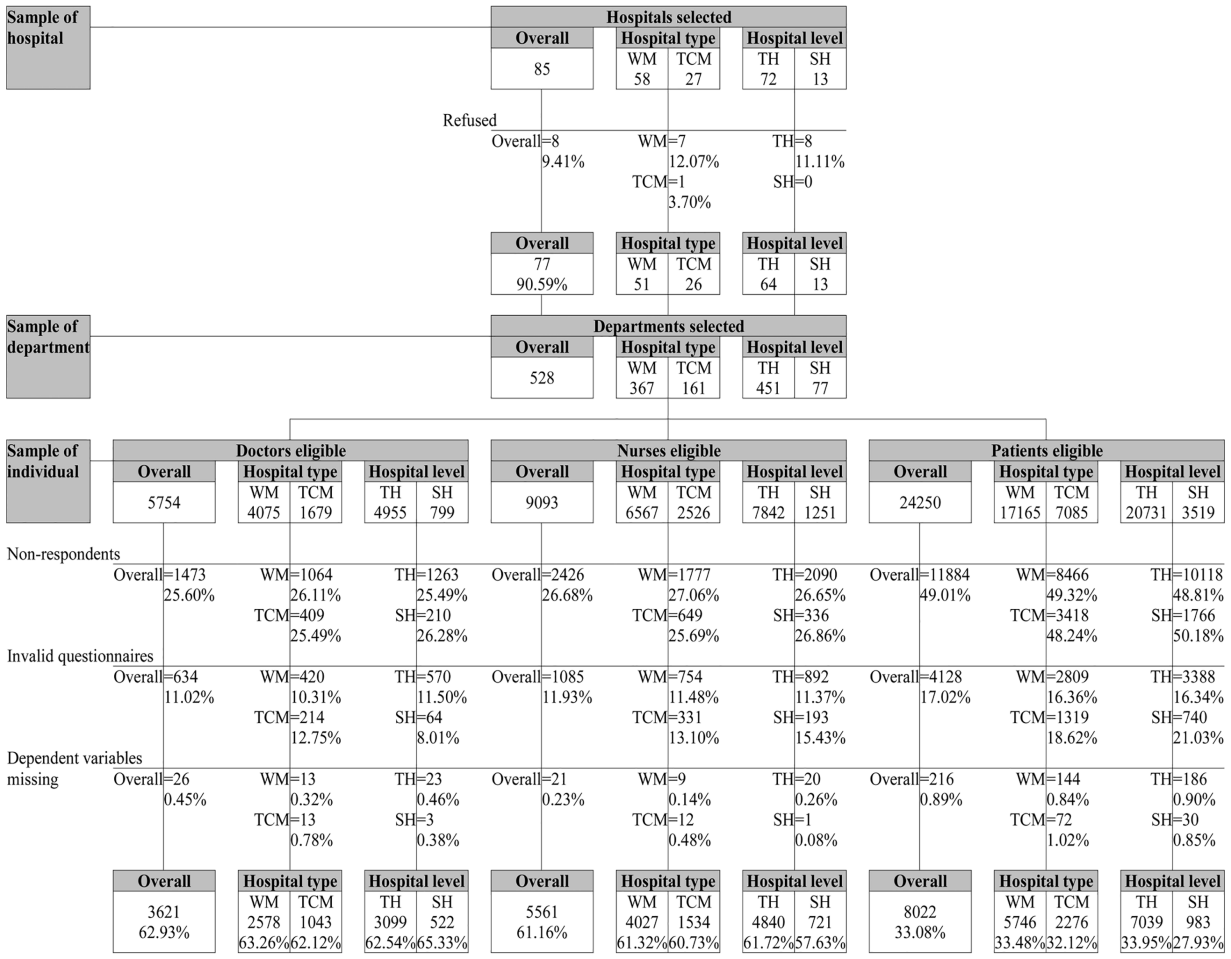
Flowchart for recruitment and response rates of the participants. WM, Western Medicine. TCM, Traditional Chinese Medicine. TH, Tertiary Hospital. SH, Secondary Hospital.

In each province, it selected one provincial hospital of Western medicine (WM) and one of Traditional Chinese Medicine (TCM), from the one or two in each area. In each provincial capital city, it selected two or three municipal people’s hospitals (usually providing WM) from the three or four available, and one municipal TCM hospital, usually the only one. convenience sampling in each province was used to select two prefecture-level cities from the six to eight in the area. In each one, it selected two WM city hospitals out of around two or three and one TCM city hospital, usually the only one. The total number of eligible hospitals was 85, of which eight refused to participate, leaving a total of 77 hospitals (90.59%). In each hospital, convenience sampling was used to select four surgical departments and four internal medicine departments (excluding obstetrics and pediatrics). A total of 528 departments were selected (Fig 1).

All full-time doctors (n = 5,754), nurses (n = 9,093) and inpatients (n = 24,250) in the 528 departments from July 2014 to April 2015 were eligible and invited to participate. Trained survey interviewers sent copies of the questionnaire in an envelope to each department, with an explanation of the purpose and method of the survey, including that participation was voluntary and their contribution would be anonymous. The survey was a self-administered paper survey, and family members were allowed to help patients fill in questionnaires. After one or two days, the interviewer returned to the department to collect completed questionnaires.

The crude response rate of doctors, nurses and patients was 74.40%, 73.32% and 50.99%. Questionnaires were given a manual check for handwriting using three trained survey interviewers, and a computer check for logical errors. The total rejected by these two tests were 634 from doctors (11.02%), 1,085 from nurses (11.93%) and 4,128 from patients (17.02%). We also excluded any participants missing the dependent variable (26 doctors, 0.45%; 21 nurses, 0.23%; and 216 patients, 0.89%). This left a total of 3,621 doctors (62.93%), 5,561 nurses (61.16%) and 8,022 patients (33.08%) (Fig 1).

Participants provided oral informed consent for interviews. The institutional review board at the Tongji Medical College, Huazhong University of Science and Technology (Wuhan, China) approved the study protocol [No. IORG0003571].

### Data collection

The survey included questions evaluating health services and health-related behaviors of doctors, nurses and inpatients. To ensure it was appropriate for our participants, we developed the questionnaire following 13 focus groups with 69 healthcare staff (including doctors, nurses and health administrators) and 73 inpatients and family members to gather information on their understanding of health services and health-related behaviors. Drawing on previous studies [28–32], we administered the draft questionnaire to small groups of doctors, nurses and inpatients in three municipal general hospitals in Hubei province, in central China. We discussed each question with them to check interpretations and modified the questionnaire if necessary. Finally, we piloted the survey in the same three municipal general hospitals, selecting one surgery and one internal medicine department in each hospital. This process uncovered difficulties, questions and concerns, such as barriers to comprehension, whether time required for completion was feasible for busy doctors and nurses, and confidentiality.

In the current study, we focused on PPR and influencing factors. PPR is an important but poorly defined topic, and research in the area has been somewhat fragmented [16,33]. According to an overview of 19 instruments assessing PPR, the instrument selection should be based on the study’s objectives and the healthcare setting in which it will be applied [20–23,33]. Although the Patient-Doctor Relationship Questionnaire (PDRQ-9) and Patient Practitioner Orientation Scale (PPOS-18) are often used for the assessment of PPR, length limits their feasibility of use in large surveys with multiple content areas [34–37]. Busy doctors and nurses are reluctant to complete long questionnaires, and we also wanted to make an identical evaluation of PPR among doctors, nurses and patients. Based on the relevant research in China [12,38], we assessed PPR using a one-item measure, the question “In general, what do you think of the current patient–provider relationship?” with three response categories: poor, fair, or good.

Our main independent variables included six organizational and two individual factors. The organizational-level variables were: hospital type (WM or TCM); hospital level (secondary or tertiary); area of specialization (surgery or internal medicine); the ratio of doctors(nurses) to ward beds. The Ministry of Health of China specifies that the ratio should not be less than 0.3 for doctors and 0.6 for nurses [39]. We divided ratios into four categories for doctors (≥ 0.3, 0.2–0.3, < 0.2) and five for nurses (≥ 0.6, 0.5–0.6, 0.4–0.5, < 0.4); doctors’ or nurses’ concerns about performance assessment [12]. We assessed this by the question: “How worried are you about your performance assessment?” with three response categories: not very much, average, or very much; patients’ perception of their healthcare cost, assessed by the question: “What do you think of your healthcare cost?” with three response categories: low, fair, or high.

The individual-level variables were: consultation levels, consulting with patients assessed for doctors or nurses by the question: “How often do you consult with your patient about their medical treatment?” and patients being consulted for patients by the question: “How often did your doctor / nurse consult you about your medical treatment?”. In each case, there were three response categories: infrequently, sometimes, and frequently; listening to patients was assessed for doctors or nurses by the question: “How often do you listen attentively to your patients?”, with three response categories: infrequently, about the same as others, frequently; and for patients feeling they can ask question by: “How difficult is it to ask your doctor / nurse something when you need?”, with three response categories: difficult, fair, and easy.

The socio-demographic variables were: gender, with the response options of female and male; age, with the response options of under 44 years old, 45–59 years old, and 60 years and over; education status, with categories of undergraduate and below, Masters and PhD for doctors; junior college and lower, undergraduate, Masters and above for nurses; and junior high school and lower, senior high school, undergraduate and above for patients; marital status, with options of married and single/divorced/widowed/other; and self-reported economic status, with options of poor, fair or good.

The effects of the market-oriented reform of medical services and the use of the Internet are indirectly reflected in some organizational and individual factors. In particular, the main pressure in performance assessment for doctors is the assessment of income generation [12], and doctors’ performance assessment shows the impact of the market-oriented reform of medical services, and patients’ perception of healthcare cost the impact of the demand side. The education level of patients shows to some extent the availability of health-related websites and other sources of medical information [40–41].

### Statistical analysis

We used binary logistic regression to calculate odds ratios (ORs) to examine the association between PPR and the independent variables. OR>1 indicates increased likelihood and OR<1 indicates decreased likelihood of perceiving poor PPR. ORs were adjusted for socio-demographic factors such as gender, age, education status, marital status, and self-reported economic status (see supplementary data). Fair and good PPR were combined to form dichotomous dependent variables. Two-sided tests were used for all the analyses, and P-values of 0.05 or less were considered statistically significant. All analyses were performed using SPSS, version 12.0 (SPSS Inc., Chicago, IL, USA).

## Results

**Fig 2:**
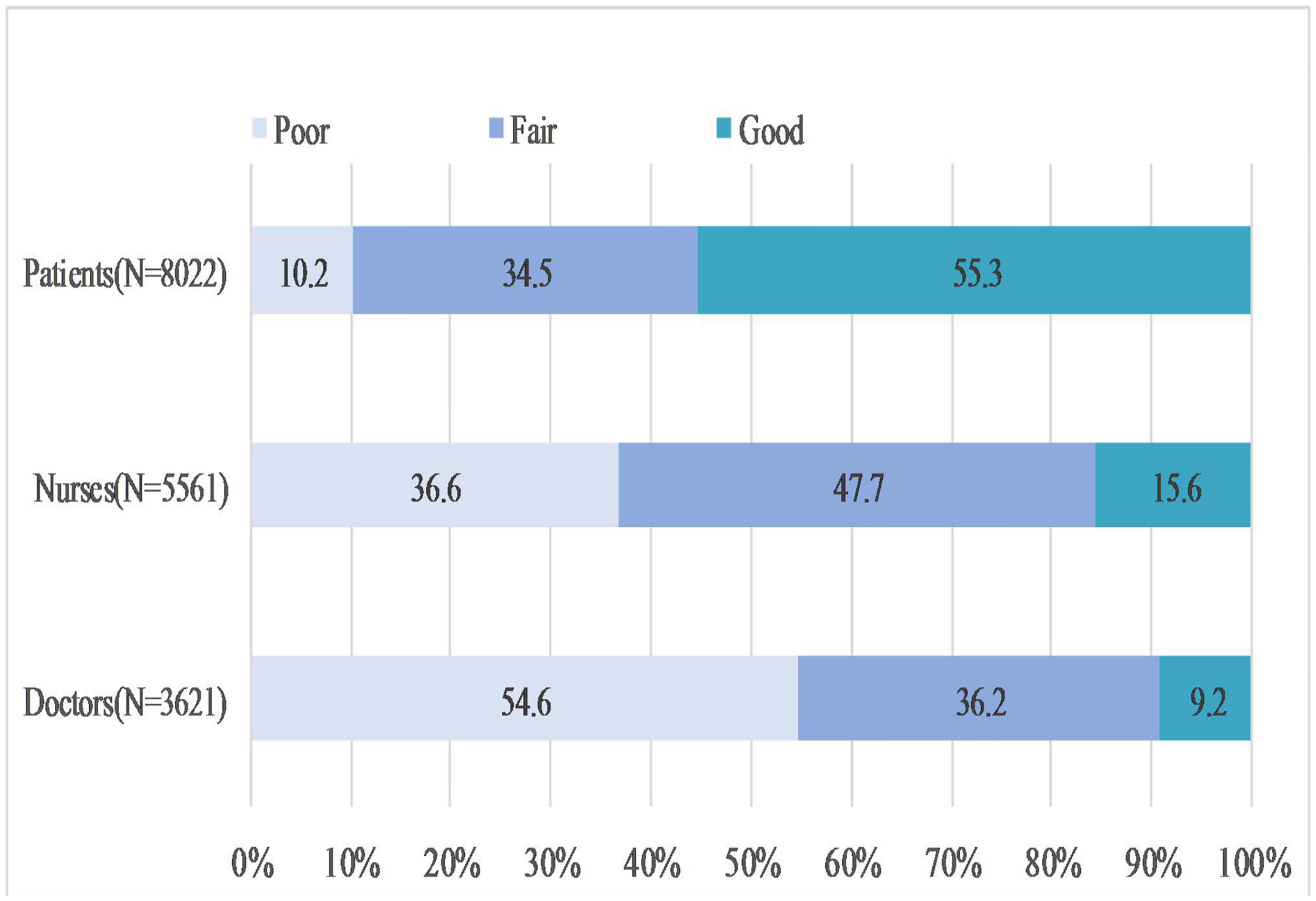
The proportion of the perception of PPR of doctors, nurses and patients.

Fig 2 shows the proportion of the perception of poor PPR among doctors, nurses, and patients. Overall, 54.6% of doctors, 36.6% of nurses, and 10.2% of patients perceived PPR as ‘poor’.

Table 1 shows the socio-demographic characteristics of the participants. The age distribution of patients is relatively balanced; those of doctors and nurses are younger, especially with nurses, which would be related to China's retirement age (in general, 60–65 for men, and 55–60 for women). Notably, self-reported economic status of doctors and nurses was generally low (38.7% and 42.2% reported with “poor”; respectively).

**Table 1:** Socio-demographic characteristic of the participants.

Variables	Doctors(N = 3621) n(%)	Nurses(N = 5561) n(%)	Patients(N = 8022) n(%)
Gender			
Female	1388(39.0)	5389(98.2)	3616(47.4)
Male	2173(61.0)	96(1.8)	4019(52.6)
missing	60	76	387
Age			
≤ 44 years old	2804(84.4)	4939(94.7)	2966(37.6)
45–59 years old	495(14.9)	277(5.3)	2247(28.5)
≥ 60 years old	24(0.7)	0	2678(33.9)
missing	298	345	131
Education status ^a^^,^^b^			
Undergraduate and below	1556(46.0)	2524(48.7)	3901(49.6)
Masters	1477(43.6)	2607(50.3)	2296(29.2)
PhD	351(10.4)	49(0.9)	1661(21.1)
missing	237	381	164
Marital status			
Single/divorced/widowed/other	945(27.3)	2295(42.8)	1538(19.4)
Married	2518(72.7)	3064(57.2)	6365(80.6)
missing	158	202	119
Self-reported economic status			
Poor	1394(38.7)	2313(42.2)	1457(18.2)
Fair	1976(54.9)	2902(52.9)	4386(54.9)
Good	228(6.3)	266(4.9)	2146(26.9)
missing	23	80	33

^a^ Education status of nurses was: Junior college and lower, Undergraduate, Masters and above.

^b^ Education status of patients was: Junior high school and lower, Senior high school, Undergraduate and above.

Table 2 shows the distribution characteristics of organizational and individual factors. Overall, the distribution of organizational factors reported by doctors, nurses, and patients are similar; and those of individual factors are quite different. Notably, only 33.7% and 9.1% of hospitals had the required ratio of doctors(nurses) to ward beds [39]. A total of 30.6% doctors and 39.6% nurses reported that they had high levels of concerns about their performance assessment, and 47.6% patients reported their perceptions of healthcare cost as “high”. High proportions (78.4% of doctors and 58.8% of nurses) reported that they “frequently” have consultations with their patients, but only 33.4% of doctors and 17.1% of nurses reported “frequently” listening to their patients. Very high numbers of patients reported difficulties asking their doctor (67.3%) or nurse (84.6%) for information.

**Table 2:** Distribution characteristics of organizational and individual factors of the participants.

Variables	Doctors n(%)	Nurses n(%)	Patients n(%)
**Organizational factors**			
Hospital type			
WM	2578(71.2)	4027(72.4)	5746(71.6)
TCM	1043(28.8)	1534(27.6)	2276(28.4)
Hospital level			
Secondary	522(14.4)	721(13.0)	983(12.3)
Tertiary	3099(85.6)	4840(87.0)	7039(87.7)
Area of specialization			
Internal medicine	1864(51.5)	2867(51.6)	4019(50.1)
Surgery	1757(48.5)	2694(48.4)	4003(49.9)
The ratio of doctors to ward beds			
≥ 0.3	1222(33.7)	-	2118(26.4)
0.2–0.3	1390(38.4)	-	3310(41.3)
< 0.2	1009(27.9)	-	2594(32.3)
The ratio of nurses to ward beds			
≥ 0.6	-	506(9.1)	527(6.6)
0.5–0.6	-	657(11.8)	762(9.5)
0.4–0.5	-	1459(26.2)	2045(25.5)
< 0.4	-	2939(52.9)	4688(58.4)
Doctors’ or nurses’ concerns about performance assessment ^c^			
Not very much	871(24.1)	1085(19.6)	377(4.9)
Average	1638(45.3)	2312(41.8)	3656(47.5)
Very much	1105(30.6)	2134(38.6)	3661(47.6)
missing	7	30	328
**Individual factors**			
Doctors' consultation			
Infrequently	149(4.1)	-	720(9.1)
Sometimes	628(17.4)	-	1942(24.5)
Frequently	2828(78.4)	-	5276(66.5)
missing	16	-	84
Nurses' consultation			
Infrequently	-	490(8.9)	625(7.9)
Sometimes	-	1786(32.3)	1888(23.8)
Frequently	-	3245(58.8)	5419(68.3)
missing	-	40	90
Doctors' listening ^d^			
Infrequently	974(27.0)	-	623(7.8)
About the same as others	1423(39.5)	-	1976(24.9)
Frequently	1204(33.4)	-	5348(67.3)
missing	20	-	75
Nurses' listening ^d^			
Infrequently	-	2488(45.0)	174(2.2)
About the same as others	-	2094(37.9)	1052(13.2)
Frequently	-	945(17.1)	6716(84.6)
missing	-	34	80

Abbreviation: WM, Western Medicine. TCM, Traditional Chinese Medicine.

^c^ Patients’ perception of their healthcare cost: Low, Fair, High.

^d^ Patients being listened to: Difficult, Fair, Easy.

Table 3 shows associations between PPR and organizational and individual variables. Organizational factors independently associated with perceptions of poor PPR included hospital type (WM vs TCM: OR = 1.25[95%CI: 1.06–1.47]) and concerns about performance assessment (high levels vs low: OR = 1.40[95%CI: 1.14–1.72]) for doctors, and concerns about performance assessment for nurses (average levels vs low: OR = 0.79[95%CI: 0.67–0.93]). Those associated with patients’ perception of poor PPR included hospital type (WM vs TCM: OR = 0.63[95%CI: 0.53–0.74]) and hospital level (tertiary vs secondary: OR = 0.65[95%CI: 0.51–0.82]). Individual factors independently associated with doctors’ and nurses’ perceptions of poor PPR were the same. They included listening to patients, marital status, education level and self-reported economic status. Those associated with patients’ perceptions of poor PPR included consultations, being listened to, marital status, education level and self-reported economic status. Doctors and nurses reporting listening to patients “frequently” had better perceptions of PPR (OR = 0.46[95%CI: 0.38–0.56] and 0.49[95%CI: 0.41–0.59] for doctors and nurses, respectively), as did their patients (OR = 0.24[95%CI: 0.18–0.31] and 0.54[95%CI: 0.35–0.84] for doctors and nurses, respectively).

**Table 3:** Adjusted associations between predictor variables and the perception of patient-provider relationships of doctors, nurses and patients in China.

Variables	Poor perception of PPR of Doctors		Poor perception of PPR of Nurses		Poor perception of PPR of Patients	
	OR(95%CI)	*p* Value	OR(95%CI)	*p* Value	OR(95%CI)	*p* Value
**Organizational factors**						
Hospital type (ref = TCM)						
WM	1.25(1.06–1.47)	.01	0.91(0.79–1.04)	.17	0.63(0.53–0.74)	<.01
Hospital level (ref = Secondary)						
Tertiary	0.82(0.65–1.03)	.09	1.05(0.87–1.26)	.64	0.65(0.51–0.82)	<.01
Area of specialization (ref = Surgery)						
Internal medicine	1.10(0.94–1.29)	.25	1.10(0.98–1.24)	.12	1.06(0.90–1.25)	.46
The ratio of doctors to ward beds(ref = ≥ 0.3)						
0.2–0.3	0.94(0.79–1.12)	.47	‐		1.03(0.85–1.27)	.74
< 0.2	0.96(0.79–1.16)	.64	‐		0.84(0.67–1.06)	.14
The ratio of nurses to ward beds(ref = ≥ 0.6)						
0.5–0.6	‐		0.83(0.63–1.08)	.16	1.04(0.70–1.55)	.84
0.4–0.5	‐		0.84(0.67–1.06)	.15	1.11(0.79–1.57)	.56
< 0.4	‐		0.86(0.69–1.07)	.17	0.97(0.69–1.37)	.88
Concerns about performance assessment (ref = Not very much) ^c^						
Average	1.06(0.88–1.28)	.52	0.79(0.67–0.93)	<.01	0.73(0.51–1.04)	.08
Very much	1.40(1.14–1.72)	<.01	0.96(0.81–1.13)	.61	0.79(0.55–1.13)	.19
**Individual factors**						
Doctors' consultation (ref = Infrequently)						
General	0.68(0.44–1.03)	.07	‐		0.75(0.58–0.97)	.03
Frequently	0.99(0.67–1.46)	.95	‐		0.68(0.52–0.88)	<.01
Nurses' consultation (ref = Infrequently)						
General	‐		0.90(0.71–1.13)	.35	1.03(0.77–1.38)	.85
Frequently	‐		0.97(0.78–1.21)	.80	0.98(0.73–1.31)	.89
Doctors' listening (ref = Infrequently) ^d^						
About the same as others	0.64(0.53–0.77)	<.01	‐		0.53(0.41–0.67)	<.01
Frequent	0.46(0.38–0.56)	<.01	‐		0.24(0.18–0.31)	<.01
Nurses' listening (ref = Infrequently) ^d^						
About the same as others	‐		0.60(0.53–0.69)	<.01	0.51(0.32–0.80)	<.01
Frequently	‐		0.49(0.41–0.59)	<.01	0.54(0.35–0.84)	<.01
**Socio-demographic factors**						
Gender(ref = Female)						
Male	1.02(0.86–1.20)	.83	1.17(0.72–1.92)	.52	0.99(0.84–1.16)	.90
Age(ref = ≤ 44 years old)						
45–59 years old	1.11(0.89–1.38)	.36	1.09(0.83–1.43)	.56	1.04(0.85–1.27)	.72
≥ 60 years old	0.99(0.39–2.50)	.98	‐		0.82(0.66–1.02)	.07
Education status (ref = Undergraduate and below) ^a^^,^^b^						
Masters	1.19(1.00–1.41)	.04	1.25(1.10–1.42)	<.01	1.44(1.18–1.75)	<.01
PhD	1.14(0.87–1.49)	.34	1.52(0.79–2.92)	.21	1.85(1.49–2.29)	<.01
Marital status(ref = Married)						
Single/divorced/widowed/other	0.54(0.46–0.65)	<.01	0.79(0.69–0.90)	<.01	0.80(0.65–0.99)	.04
Self-reported economic status (ref = Poor)						
Fair	0.54(0.46–0.64)	<.01	0.63(0.55–0.71)	<.01	0.69(0.57–0.85)	<.01
Good	0.34(0.24–0.47)	<.01	0.56(0.41–0.77)	<.01	0.52(0.40–0.66)	<.01

Abbreviation: WM, Western Medicine. TCM, Traditional Chinese Medicine.

^a^ Education status of nurses was: Junior college and lower, Undergraduate, Masters and above.

^b^ Education status of patients was: Junior high school and lower, Senior high school, Undergraduate and above.

^c^ Patients’ perception of their healthcare cost: Low, Fair, High.

^d^ Patients being listened to: Difficult, Fair, Easy.

## Discussion

To the best of our knowledge, this study provides the first national evidence in China about PPR that assesses the views of doctors, nurses and patients. Our findings that doctors have the lowest perceptions of PPR, empirically verifying its severity in China [6–8]. Of the organizational factors, doctors of TCM reported better perceived PPR than those of WM. More concerns about performance assessment was associated with doctors’ perception of poorer PPR. Patients of tertiary and WM hospital reported better perceived PPR than those of secondary and TCM hospital, respectively. Additionally, lower doctors(nurses) to ward beds ratio was not associated with bad perception of PPR in any of the respondent groups. Of the individual factors, more consultation reported by doctors and nurses was not associated with their better perception of PPR. However, more consultation only of doctors was associated with better patients’ perception of PPR. Listening to patients was associated with better perception of PPR in all of the respondent groups.

The effects of hospital type on doctors’ perception of PPR might be related to the severity of their patients’ illnesses. Over the past few decades, TCM has been in decline, and has had to learn from WM [38]. Nowadays, WM is at a distinct advantage over TCM, in terms of both the qualifications of healthcare staff and the number of hospitals, so the vast majority of patients prefer to attend a WM hospital, especially when they suffer from serious or life-threatening illnesses. The mortality risk among patients of WM hospitals are therefore often higher [42]. When patients die, and families still have to pay medical fees, they may be angry, and blame health staff. Extreme violence against healthcare staff has been seen in recent years [6]. Doctors affiliated with WM hospitals are therefore likely to have worse perception of PPR. This may also reveal a lack of awareness about complexity of diseases and the limitations of medical care among patients [43]. Patients with more serious illnesses need better PPR, but may, our results suggest, be least likely to develop those relationships. It is therefore necessary to improve patients’ understanding of the limitations of medicine.

Performance assessment of doctors is a reaction of healthcare provider organizations to the market-oriented reform of medical services. In the past 20 to 30 years, government financial subsidies for public hospitals have been inadequate in China [44]. More recently, however, benefits have been seen from the new medical reforms starting in 2009, with increasing government financial subsidies, although these only accounted for an increase in total income of public hospitals from 7.81% in 2008 to 8.15% in 2012 [45]. The vast majority of the income of public hospitals is therefore self-generated, relying heavily on charges for drugs and medical examinations. These income-generating activities are a key indicator in the performance assessment of doctors [12,38]. Doctors are therefore required to consider not only the complexity of diseases, but also the requirement to generate income. Unlike fully market-oriented private hospitals or profit-driven corporate models for healthcare delivery, income generation in public hospitals in China is usually passive. Our findings suggest that an average level of worry among doctors about performance assessment has no significant effect on perceptions of PPR. Only those with high levels showed any significant negative effect on PPR, showing that doctors have to find a balance between income generation and doing the right thing for their patients. This suggests that moderate rather than excessive (perhaps the golden mean) performance assessment for doctors will be better for PPR.

China’s secondary and tertiary hospitals are usually general hospitals, with higher overall levels of medical treatment in tertiary hospitals. Patients who attend tertiary hospitals usually suffer from more serious conditions, so there may be more potential for medical disputes. We found, however, that there was no difference in perceptions of PPR among doctors working in different levels of hospital. This may suggest that as the result of a chilling effect [46], doctor affiliated with secondary hospitals would have the similar perception of PPR with those affiliated tertiary hospitals.

The effects of the type and level of hospitals on patients’ perception of PPR may be related to the core needs of the patients. The level of medical treatment in WM and tertiary hospitals is likely to be higher, with patients having more needs. These patients may therefore have a better perception of PPR, even with higher fees and more difficulty seeing doctors. There were no significant effects of medical fees on patients’ perception of PPR. This may be for two reasons. First, patients with higher levels of need recognize that they need to pay more. They also believe that life is more important than money. The second reason is the universal coverage of health insurance, which eases the financial burden on patients [43]. Nearly 30 years of market-oriented reform of medical services has also enabled patients to accept medical fees as the norm.

The ratios of doctors(nurses) to ward beds is, in many cases, far below the level stipulated by the Ministry of Health of China [39]. This suggests high workloads among staff. On the surface, lower ratios mean busier staff, which would lead to tension, and perhaps lower PPR. However, our survey showed no significant effects of the ratio on PPR. This may be for two reasons. First, lower ratios mean higher incomes for staff. They are therefore unlikely to complain or perceive problems with PPR, even when working hard. Second, patients in China have a completely free choice of hospital [47,48], and most favor general hospitals providing higher levels of care, regardless of the higher fees or the levels of crowding [49]. Providers may see having lots of patients as a measure of patients’ approval, and even an honor. This may also be why these ratios have no effect on patients’ perceptions of PPR. In other words, patients’ perceptions of PPR do not rest on the cost or difficulty of seeing doctors, but whether they can be successfully treated.

The difference between doctors and nurses in effect of hospital type on perceptions of PPR is probably related to their different roles. The difference in the effects of performance assessment is probably related to the differences in content of performance assessment for nurses and doctors. Performance assessment for doctors is mainly focused on income generation. For nurses, however, performance assessment mainly focuses on quality of care [50]. For nurses, therefore, higher levels of worry about performance assessment would probably improve PPR, although this effect might be countered by the severity of the performance assessment. These differences also suggest that it may be important to use an integrated system of performance assessment for doctors and nurses.

Improved patient-provider communication should improve PPR [13,14]. We divided communication into consultation with patients (doctor/nurse-based) and listening to patients (patient-based) [51]. The differences between doctors, nurses and patients in the effect of consultation on PPR is possibly related to the content discussed. Consultations often focus on disease status and changes, treatment options, medical risk and informed consent [52]. There is an information asymmetry between patients and healthcare staff. Consultation, especially with doctors, ensures that patients are kept informed, and will improve their perceptions of PPR. For healthcare staff, however, consultation with patients is routine work, and has no significant impact on their perception of PPR. Unlike consultation, listening focuses on patients, and also considers their psychological and social needs [14, 15,53]. Recent increases in the incidence of chronic non-communicable diseases mean that patients’ need to be listened to is greater than ever [54]. Unfortunately, it is not clear that the behavior of doctors and nurses has also changed to address these needs, and the required behavioral transformation lags behind the multi-dimensional health needs of patients.

The effects of economic status on doctors and nurses’ perceptions of PPR is probably related to market-oriented reform of medical services. If doctors or nurses have better economic status, the pressure and psychological burden of income generation is less, so their perception of PPR is better [55]. Patients with higher economic status tend to pay more attention to their illness and work with healthcare staff to manage their treatment [56,57]. Their perceptions of PPR are therefore likely to be better. The effect of patients’ educational levels on perceptions of PPR is probably related to access to medical information. This is consistent with other reports that access to medical information has a negative influence on perceptions of PPR [9]. Patients’ educational level and access to sources of health information is may not mean that their judgment about their diseases and treatment is correct, which may lead to conflict with healthcare staff. More importantly, if highly educated patients have worse perceptions of PPR, this possibly has a ‘trickle-down’ effect on others, and may aggravate the deterioration of PPR. Although patient-provider communication was regarded as one component of PPR in some studies, more studies regarded it as an influencing factor of PPR (i.e., communication is a process, and PPR is an outcome) [16,33]. Especially, less patient-provider communication or poor patient-provider communication skill could be related to poor PPR perceived by patients and providers, which are generally consistent with the results of this study. As mentioned above, our study differentiated two aspects of communication: consultation with patients (providers based) and listening to patients (patients based) [51], and showed the difference of their effects on PPR.

Continuity of care was not involved as an individual influencing factor in this study mainly with the suggestion of the instrument selection of PPR in this study. In China, patients can select freely nationwide hospital or doctor, as buying clothes in mall. Sometimes they would see a few hospitals at the same time; even at the same time see a few doctors in a hospital, especially they are suffering from serious illness. Additional, China is almost universal medical insurance, and also can achieve national networking, the use of personal medical insurance card is not limited to hospitals or doctors, but to the proportion of reimbursement of medical expenses varied with hospital levels and areas. Therefore, continuity is very weak in China, especially for patients in general hospitals.

Notably, organizational and management measures are considered important for promoting continuity of care and doctors’ communication skill training [16]; however, organizational factors themselves have rarely been examined in previous studies, and the corresponding evidence and recommendations are also lacking. Our findings, especially the impact of performance assessment, the ratio of doctors (nurses) to ward beds and hospital characteristics, would not only provide the evidence for organizational management measures promoted PPR, but also provide a reference and comparison for follow-up study.

Among the social and demographic factors, the previous studies showed that patients with poor economic status report lower PPR [38, 58,59], which is consistent with the results of the current study. Notably, our findings would broaden the impact of economic status, namely the impact on doctors and nurses perceived PRR. Furthermore, the impact of self-reported economic status may be more important than those of education status, especially for doctors and nurses. Additional, compared to the participations with married, whether doctors, nurses or patients, those unmarried would report better PPR, which seems to be of little concern in prior studies. As a specialized form of human relationship [60], individual perceived PPR might be transferred or replaced with their marriage relationship, which would be worthy of follow-up study.

### Strengths and limitations

Our study has several strengths. First, the study data are collected from a nationally representative sample in China. Second, we conducted an analysis with organizational and individual factors, extending the existing studies that focused primarily on individual factors. Third, the study examined perceptions of PPR among doctors, nurses, and patients simultaneously. Limitations include, first, the response rate was low. We did not use any incentives or persuasion to improve participation, as this would have increased potential information bias. Similarly, low response rates have been seen in other national surveys of doctors, and some have shown no significant differences between responding and non-responding doctors [61–63]. We were, however, unable to report on this, as we had no data about non-respondents. Our results for doctors’ and inpatients’ perceptions of PPR, however, were similar to another survey using the same dependent variable with a smaller sample of 500 healthcare staff and 510 patients, and a high response rate (98.4% and 99.61%) [11]. Doctors’ demographic characteristics in our study were similar to those of other studies with a high response rate (89.3% to 93.6%) in China [64,65]. Second, our study relied on cross sectional data, which could not definitively establish causality. Besides all *p* values should be regarded as exploratory rather than hypothesis testing, and moderate *p* values should be interpreted cautiously. Third, we excluded obstetrics and pediatrics in departments sample, which may bring some selection bias.

## Conclusion

In a nationally representative sample, the deterioration of PPR were serious with doctors’ perceptions in China. Although our findings require validation in different organizational settings given the likely variability of these associations across systems, our results suggest that implementing moderate levels of performance assessment, promoting the level of medical treatment, and broadening doctors/nurses training contents with listening to patients, may benefit to promoting PPR.

## Supporting information

S1 File Supplementary data of figure 1PM. (XLSX)

S2 File Data. (XLSX)
